# The Beauty and the Beast

**DOI:** 10.1523/ENEURO.0142-21.2021

**Published:** 2021-04-29

**Authors:** Christophe Bernard

How can we evaluate the positive impact that visionary people can have on science? Should we support funneling large amounts of money to big projects? Both questions arose when I watched Noah Hutton’s film, *In Silico*.* I invited several prominent scientists to comment on the film’s main topic, modeling the human brain, in a special collection for *eNeuro*.

The film tells the 10-year journey beginning with Henry Markram’s 2009 TED talk (https://www.ted.com/talks/henry_markram_a_brain_in_a_supercomputer), where he announced that the brain could be modeled within 10 years in a supercomputer. The scientific initiative began in 2005 at École Polytechnique Fédérale de Lausanne when Markram founded and directed the first scientific initiative toward his goal: the Blue Brain Project (https://www.epfl.ch/research/domains/bluebrain/). Then, in 2012, the European Union selected the Human Brain Project (https://www.humanbrainproject.eu/en/), also then led by Markram, as one of two flagship programs to be awarded €1 billion over 10 years. Modeling the human brain was one objective (Destexhe, 2021). For a scientific and historical perspective on the Blue Brain Project and the Human Brain Project, you can refer to Yves Frégnac’s commentary (Frégnac, 2021).

As indicated by Eve Marder (Marder, 2021), one cannot but fall for the beauty of the brain. This happened to me when I saw a Golgi staining of a Purkinje cell during my first visit to a neuroscience lab. My first research project was on a computer model of the cerebellum. I then discovered the beast: by tweaking the model parameters, I could make it behave as I wished. Other commentaries (Jirsa, 2021; Marder, 2021) mention that because of degeneracy (there are multiple solutions to the same problem), and because many important parameters were omitted (Fairhall, 2021), the probability to construct a working brain via the inclusion of parameters measured from different animals would be close to 0. As a scientist, my original take on Markram’s endeavor is best illustrated by Vineet Tiruvadi’s statement: “If you start with the wrong framework then the ability to do complex analyses may seem like it’s giving insight, but what you're mostly doing is studying how wrong your framework is” (Tiruvadi, 2021).

But my point here is not to criticize Markram’s approach. Rather, I would like to open a discussion on science. History is full of individuals with vision and the will to impose it, from prophets to scientists. Perhaps, mankind’s suffering is what allows the emergence of such individuals; leaders answer a need, such as the promise of a better world (prophets) and the promise of solutions to big questions (scientists at a standstill in their quest to answers). There are individuals I, as a scientist, admire for their vision or insight (e.g., Santiago Ramon y Cajal). Would I meet someone with the potential to make a paradigm shift, I would gladly follow their steps. Perhaps they are already among us, but either they are not vocal enough or their voice is muffled by our attitude, which tends to disregard what is not within the accepted-by-most framework of what neuroscience is or should be.

This framework is both necessary and an impediment to progress. Seasoned scientists know the rules of the game, rules that are internally generated (by us). If you build a research project following the rules, it provides a route to funding. If the topic is fashionable, you will publish in top-tier journals.

Technological developments play a key role in driving the framework in which neuroscience is done. I remember the time when knowing the genome would be the answer to all our questions. Geneticists promised that this knowledge would solve human diseases. Other leaders promised that the ability to manipulate the mouse genome would provide final answers to the role of all proteins and to brain function. Similar claims are being made with optogenetics and chemogenetics. These technological advances did not provide a final answer. Rather, in my mind, they reveal how much more complex the brain is than we thought.

Computer modeling is a powerful technique. It has been successfully used in many science fields. Thus, modeling the brain is a logical step to take to understand it. If you want to model the brain in all its details, you will need a supercomputer. Markram proposed that this is possible with the existing technology or those under development. Many individuals had a similar idea before. For example, Roger Traub has been making heroic efforts to understand seizures using a combined theoretical and experimental approach. He was given access to IBM’s supercomputers. Traub is anything but vocal. He did not build a research center; he did not gather many scientists around him. He has always been working with a small number of neuroscientists. If he had been given €1 billion when he started to make models 30 years ago, would we have solved seizure mechanisms? There is no way to know. Then again, Viktor Jirsa makes a strong point emphasizing the fact that no one individual is given €1 billion; the amount was awarded to a consortium of scientists and subprojects (Jirsa, 2021). Markram was obviously very persuasive in both getting funding and gathering scientists around him to follow his vision (although the former may play a key role in the latter).

Do we need charismatic leaders? It is easy to say that a leader’s worth can be evaluated by a final quantifiable output. That is too easy. We will never know quantifiably because there is no way to know what would have happened otherwise. My opinion is that we may need them. They can boost a field and, if they fail, we can learn from it. One clear result of both the Blue Brain Project and Markram’s part in the Human Brain Project is a large amount of experimental data curated and made available to the community. As discussed by Adrienne Fairhall, the sharing of large datasets with the community is also one important outcome of the projects run by the Allen Institute (Fairhall, 2021). Such big projects have the advantage to federate and organize neuroscience in a coherent manner, which is more productive. Of course, one has to be careful and ensure that the vision does not become dogmatic, which then produces more bad than good.

If vision is the Beauty, money is the Beast. Perhaps a mathematician has already invented specific mathematics that explains the brain (instead of our continuous struggle to adapt concepts developed for physics and other fields), at the cost of few sheets of paper and pencils. But the current state is that neuroscience needs more and more money, and more complex and costly machines to explore the brain. Money is limited, and we, scientists, are very numerous and competing to get a share of it to do our research. The Blue Brain Project and the Human Brain Project are swallowing a large amount of money. If you are in one of these, you may be well financed. But if you are not, you may think that you would have done better with the same amount of money.

The European Union gave €1 billion to two projects, but there were six finalists. What would have happened if the projects on individual medicine or on robot companions for citizens had been funded? Would health care be much better? Would the quality of life of the elderly be improved? Again, there is no way to tell because the Human Brain Project won one of the two grants. We will see what it will deliver.

We should also reflect on the way the scientific priorities are defined (Frégnac, 2021). The European Union requests that scientists tell them which topics should be funded, which is good. I have answered such calls. However, the scientists who manage to organize themselves can act as lobbyists, and if you happen to be highly vocal and well introduced in the European Union administration, your topic may be more likely to be chosen. Where the money ends up may thus not be determined by what is more interesting or important scientifically, a risk inherent to this type of procedure. As Fairhall explains in *eNeuro*, the Brain Initiative worked differently, and other big projects started with a highly focused question (Fairhall, 2021).

Interestingly, the Human Brain Project initiative triggered other projects worldwide, as if understanding the human brain was a race one country or federation must finish first to win. To win what?

The analogy with the race to the moon is often made. Sending humans to the moon and bringing them back was successful thanks to a lot of money and people working toward the same goal. But the analogy does not make sense. The race to the moon was a technological problem. The conceptual framework was known to all. The laws of physics were available to plan trajectories, to calculate thrust to send a certain mass into orbit, to design engines, life support equipment, etc. Problems were tough, a lot of innovation was needed, but there was no theoretical barrier. In neuroscience, there is no such conceptual framework: there is no brain theory, and this is our barrier.

Arguably, sending people to the moon was useless, just a gigantic ego manifestation to beat the Russians. NASA claims that going to the moon produced huge benefits to us (https://www.nasa.gov/directorates/spacetech/feature/Going_to_the_Moon_Was_Hard_But_the_Benefits_Were_Huge). However, when you read the list, those benefits appear meager. Since John F. Kennedy’s 1962 speech, malaria may have killed the equivalent of the United States population. Would it have been better for humanity to spend the same amount of money and mobilize such human resources to eradicate malaria?

As for the Human Brain Project, of course, as a scientist, I am selfishly happy to benefit from it. Perhaps such projects are necessary to prevent and treat all neurologic disorders, a very important problem for humanity. But are we truly investing in our future? Will the generations to come praise us for our insight, or will they condemn us because we chose not to dedicate all our money and efforts to find a solution to save the planet and prevent the extinction of so many species?

Some neuroscientists like to describe some dynamic phenomena in the brain as bifurcations, i.e., sudden qualitative changes in a system’s behavior. Did we go through one such bifurcation via Henry Markram’s vision and the funding of big brain projects? Or is it just a hiccup in neuroscience history?

Who will release the princely brain from its beastly curse? Gabrielle-Suzanne Barbot de Villeneuve did not write about that. This should be our mission, together.

*In Silico will be available beginning April 30, 2021, at https://insilicofilm.com/.
